# Meta-analysis of Selective Serotonin Reuptake Inhibitors (SSRIs) Compared to Tricyclic Antidepressants (TCAs) in the Efficacy and Safety of Anti-depression Therapy in Parkinson's Disease(PD) Patients

**Published:** 2014

**Authors:** Bao-Yue Qiu, Jun-Xiao Qiao, Jiang Yong

**Affiliations:** Department of Medical Engineering, General Hospital of the Second Artillery, Beijing 100088, China.

**Keywords:** Meta-analysis, Selective serotonin reuptake inhibitors (SSRIs), Tricyclic anti-depressants (TCAs), Efficacy, Complication

## Abstract

To assess the efficacy and safety of selective serotonin reuptake inhibitors(SSRIs) and tricyclic antidepressants(TCAs) in treatment of Parkinsonian depression.

A computer-based search was conducted in the databases of PUBMED, MEDLINE, EMBASE and CochraneControlled Trails Register up to December 2011. The random controlled clinic trials about SSRIs and TCAs in treatment of Parkinsonian depression were collected. Statistical analysis was completed using Review Manager, version 5.0.

Five clinic controlled trials were identified for this meta-analysis. There was no significant statistical difference in the response rate of treatment [RR 0.95, 95%CI (0.78, 1.16)] and Hamilton depression score [RR -2.54, 95%CI (-5.35, 0.26)] between two groups. In term of complications, no statistical difference was observed in the insomnia rate between two groups [RR 0.82, 95%CI (0.24, 2.84)]. Moreover, the incidence rate of xerostomia [RR 0.21, 95%CI (0.07, 0.65)] and constipation [RR 0.12, 95%CI(0.02, 0.63)] was lower in SSRIs group rather than those in TCAs group.

In general, SSRIs and TCAs have comparable efficacy and equal acceptability in treatment of Parkinson’s disease-induced depression. However, SSRIs are superior to TCAs in the terms of xerostomia and constipation.

## Introduction

Parkinson’s disease (PD) is a common neurodegenerative disorder in wrinkly and elderly people.It is manifested clinically by impaired motor functions(bradykinesia, tremor, rigidity, flexed posture, postural instability, and freezing of gait) as well as non-impaired motor functions (depression, dementia, drug-induced psychosis, impulsion, and sleep disorders)(1). Of which, depression is the most common symptoms of non-impaired motor functions in PD, accounting for 40% to 50% occurrence (2). Among these patients, only 20% to 25% of the PD patients are provided with nursing and anti-depression therapy(3). 

Depression symptom could appear in each period of PD, significantly affecting the life quality of patients.Tricyclic antidepressants(TCAs) have the major pharmacological treatment for depression, which inhibit presynaptic norepinephrine or serotonin uptake. However, the TCAs can cause anticholinergic, antihistaminergic and cardiotoxicun wanted effects which are related to their action on muscarinic, histamine, adrenergic receptors and cardiac Na^+^and Ca^2+^channels(4). Additionally, the selective serotonin reuptake inhibitors (SSRIs) have a high affinity to serotonin uptake but a low affinity to noradrenaline uptake sites and neurotransmitter receptors, which have become the first line of rationally designed therapeutic agents in psychiatry due to the little side effect and good safety(5).

Although there has been a large number of studies performed that report the efficacy and safety of SSRIs and TCAsfor the treatment of depression, most are either retrospective case series or single-center clinical trials. The exacts lot of SSRIs and TCAs in the therapeutic strategy of PD-induced depression still remainscontroversial. To address existing uncertainties, we performed meta-analysis that allows pooling of data from all head to head trials and the results will help clinicians in selecting agents for the treatment of PD-induced depression

## Experimental

*Search strategy*

To identify and retrieve all potentially relevant literature describing the outcomes of SSRIs and TCAs for treatment of Parkinsonian depression, we performed a literature search in PubMed, MEDLINE, EMBASE, Cochrane Controlled Trails Register (CCTR), and Google scholar up to the end of 2011. The search terms were: Parkinson’s disease AND depression AND (selective serotonin reuptake inhibitorsOR paroxetine OR fluoxetine OR citalopram OR sertraline OR escitalopram) AND (Tricyclic antidepressants OR imipramine OR amitriptyline OR doxepin OR clomipramine OR desipramine OR nortriptyline).

*Identiﬁcation of studies*

Studies that met the following criteria wereeligible for inclusion in the meta-analysis:(1) primary documents published at home and abroad; (2) randomized clinical trial (RCT) and/or clinical control trial (CCT); (3)studies that reported the year of publication; (4) studies that stipulated the sample size; (5) studies that clearly elucidate the diagnostic criteria of PD; (6) SSRIs and TCAs therapy involved in treatment of PD; (7) studies that reportedthe comparison of HAM-D score, life quality and other outcomes; (8)data collected scientifically; (9) data analyzed correctly.

We excluded trials in which subjects suffered from other mental disorders since the principle aim of this study is to evaluate the outcomes of Parkinsonian depression treatment. Trials thatincluded formal psychotherapy in combination with antidepressants were excluded but those that randomized psychotherapy against antidepressants and analyzed data separately were included. Studies comparing SSRIs with older antidepressants which do not block monoamine reuptake were excluded in the meta-analysis as they have varying different pharmacological mechanisms. Newer antidepressants such as nefazodone, venlafaxine and mirtazapine were also excluded because their heterogeneous pharmacology and remain relatively infrequently used. 

*Literature evaluation and data extraction*

The quality of studies was evaluated according to [Cochrane reviewer’s handbook]as follows: (1) the random method described or not; (2) the allocation concealment method employed or not; (3) the double-blind method employed or not; (4) lost follow-up or dropout reported or not; intention to treat analysis(ITT) conducted or not; (5) baseline was consistent or not. The trials were also assessed according to quality scoring reported by Jadad *et al.*(6). Jadad scoreof more than 3 indicates the high quality of trials. 

Two independent investigators assessed eligibility and abstracted the data. When specific variables were not reported within a given study, the authors of the paper were contacted to obtain the missing data. Discrepancies between reviewers were resolved by discussing with the third investigator.

*Statistical analysis*

Outcomes including response rate, insomnia incidence, xerostomia and constipation were pooled by the Mantel-Haenszel method for risk ratios (RR) with 95% CIs. In addition, the absolute scores of Hamilton Rating Scale for Depression (HAMD) were converted into a common unit by calculating the weighted standardized mean difference (SMD) with 95% CIs. Standardized effect sizes were derived by dividing the mean difference in HAMD scores between SSRIs and TCAs subjects of each individual trial. When the RR and SMD were not available in the source papers, authors were contacted.

Statistical heterogeneity of the results from individual studies was examined using Cochran’s Q test(7) and I^2^index (8). If significant heterogeneity was observed (P≤0.10; I^2^>50%), a random effects modelwhichassigns a weight to each study was used to poolthe results together. If homogeneity was identified (P>0.10; I^2^<50%), a fixed-effects model was used (a common underlying effect is being pooled).

All analyses were carried out using Review Manager, version 5.0 for Windows. P < 0.05 was regarded as statistically difference.

## Results

A total of 33 trialscomparing SSRIs and TCAs in the treatmentof PD-induced depression were screened out by the initial search strategy. Of which, 20 literature reviews and/or commentary literature, 3 studies that reported the effect of dopamine and other drugs, and 6 studies that did not presentedrelated outcomes were excluded from the meta-analysis. Finally, 5 trialsmet our criteria were selected into this meta-analysis(-).

*Characteristics of studies*

The author, year of publication, journal published, type of study, number of patient, method of treatment, time of follow-up and quality scoring of study were listed in Table 1 and Table 2. A total of 5 s tudies(-)selected in this meta-analysis were prospective clinic random controlled trials and published from 1996 to 2009. The number of participants was ranged from 77 to 31, including 106 patients in the SSRIs group and 116 patients in the TCAs group. Five literatures(9-13)achieved a Jadad score of 5. 

**Table 1:** Quality analysis of studies included

**Study,publication date**	**Journal**	**Type of study**	**Number of patients**	**Duration of study**	**Jadad score**
Antonini 2006	MovDisord	RCT	31	12 weeks	5
Devos 2008	MovDisord	RCT	32	4 weeks	5
Menza 2009	Neurology	RCT	35	8 weeks	5
Rabey 1996	Neurology	RCT	47	16 weeks	5
Serrano-Duenas 2002	Rev Neurol	NA	77	12 months	5

**Table 2:** Baseline of studies included

**Study, publication date**	**Drug name**	**SSRIs** **dose (mg/d)**	**Subjects**	**Drug name**	**TCAs** **dose (mg/d)**	**Subjects**
Antonini 2006	Sertraline	50	16	Amitriptyline	25	15
Devos 2008	Citalopram	20	15	Desiprmine	75	17
Menza 2009	Paroxetine	28.4	18	Nortriptyline	48.5	17
Rabey 1996	Fluvoxamine	78	20	Amitriptyline	69	27
Serrano-Duenas 2002	Fluoxetine	27.3	37	Amitriptyline	35.2	40

*T* *he response rate*

**Figure 1:**
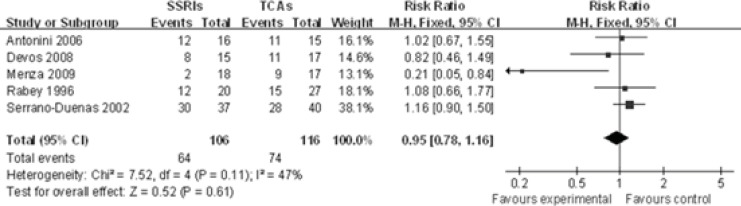
Comparison of the response rate of PD treatment between SSRIs group and TCAs group

The response rate of five individual SSRIs compared with a TCAs comparator was investigated(-). TheCochran Q test and I^2 ^index for heterogeneity (P = 0.11, I^2^=47%) indicatedthat the studies are homogenous and could be combined. Hence, the fixed effects for the summaryof RR of studies have been applied. In the analysis of all studies, there was no difference in response rate between the individual SSRIs and their TCA comparators [RR 0.95, 95% CI(0.78, 1.16), P=0.61], suggesting a comparable effect of SSRIs and TCAs in terms of response rate (Figure 1).

*HAMDscore*

**Figure 2:**
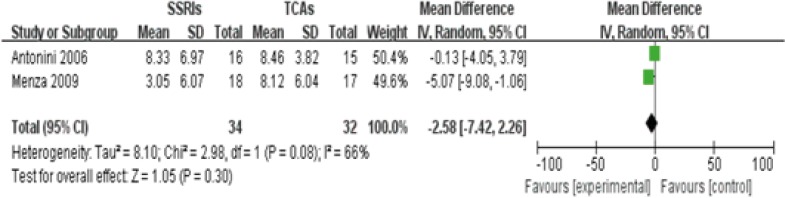
Comparison of difference value in HAMD score of PD treatment after 4 weeks between SSRIs group and TCAs group

Two studies(9, 11) reported different value of HAMD score between SSRIs and TCAs group in the treatment of PD-induced depression after 4 weeks, containing 34 patients in SSRIs group and 32 patients in TCAs group. The heterogeneity existed among these two studies (P=0.08<0.1, I^2^=66%), thus, a random-effects model was employed to analyze these studies. No statistical difference in HAMD score between SSRIs group and TCAs group was observed[Z score=1.05,95%CI(-5.35, 0.26), P=0.08].This finding indicates that SSRIs and TCAs have equal effects on reducing the HAMD score among patients with Parkinsonian depression(Figure 2).

*I* *nsomnia*

**Figure 3:**
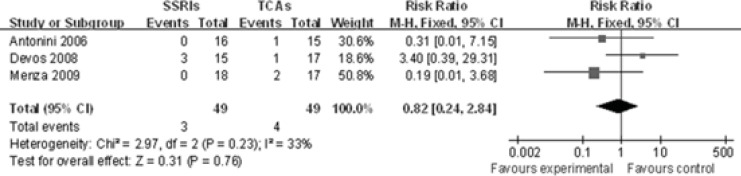
Meta-analysis of insomnia rate between SSRIs group and TCAs group

All-cause insomnia data were available from 3 trials(-). Becauseno heterogeneity among these three studies was identified (P=0.23, I^2^=33%)for the overall comparison, a fixed-effects model was used to analyze these studies. There was no statistical difference between these two groups[RR 0.82, 95%CI(0.24, 2.84), P=0.76], indicating that SSRIs are not superior to TCAsin the occurrence of insomnia (Figure 3).

*Xerostomia*

**Figure 4:**
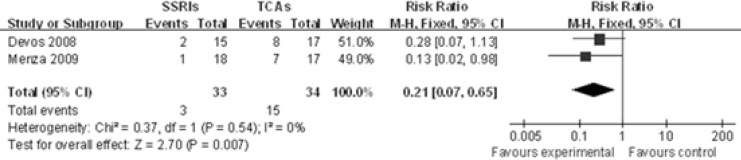
Meta-analysis of xerostomia rate between SSRIs group and TCAs group

Two studies(10, 11)showed the xerostomia rate between SSRIs group and TCAs group in PD treatment. The incidence of xerostomia was significantly different between SSRIs and TCAs[RR 0.21, 95%CI (0.07, 0.65), P=0.007], using a fixed effects model due to the lack of significant heterogeneity in the data collected (P=0.54，I^2^=0%). This indicates that the occurrence of xerostomia favors SSRIs group rather than TCAs group (Figure 4). 

*Constipation*

**Figure 5:**
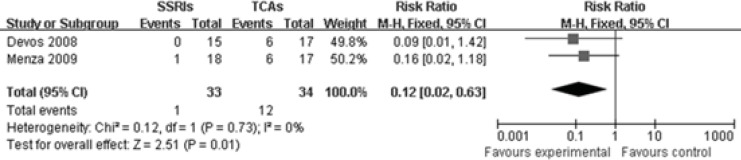
Comparison of constipation rate between SSRIs group and TCAs group

Two studies(10, 11) reported comparison of constipation rate between SSRIs group and TCAs group. The heterogeneity did not exist among these two studies (P=0.73, I2=0%),so a fixed-effects model was used. There were markedly statistical difference between these two groups[RR 0.12, 95%CI(0.02, 0.63), P=0.01], illustrating that SSRIs are superior to TCAs in reducing the constipation rate among patients with PD-induced depression (Figure 5).

## Discussion

The main objective of this meta-analysis is to providereliable assessments of the major benefits and risks ofSSRIs and TCAs therapy in management of Parkinsonian depression. We aimed tominimize both systematic and random errors by bringing together individual participant data from alleligible large randomized trials comparing SSRIs therapy versus TCAs therapy, and by prespecifying the main analysis such as the outcomes of the response rate, HAMD score, insomnia, xerostomia and constipation. The number included in the meta-analysis was not excessive, partly due to the fact that many of the trials failed to meet our inclusion criteria. Some studies reported different interventions to treat depression, while others used different endpoints to measure the effect size of antidepressants. Finally, five RCT studies with the Jadad score of 5 were pooled in the evaluation of efficacy and safety of SSRIs compared with TCAs.

The results of this meta-analysis help toclarify that there was no difference in acceptability as measured by the response rate and HAMD score between SSRIs and TCAs. The response rat and HAMD score are two major indicates to evaluate the efficacy of antidepressants. Indeed, successful treatment can be defined as a decrease in HAMD score to less than 8(14). Several previous studies have reported the similar results that SSRIs and TCAs generally have an equal efficacy, although SSRIs have superior tolerability and safety benefits over older TCAs(-).

Two previous meta-analysis comparing SSRIs and TCAs have suggested that SSRIs are better tolerated than TCAs as measured by total dropout rate due to the side effects (17, 18). However, the term of side effects was not listed in these two studies. Our present meta-analysis investigated side effects including insomnia, xerostomia and constipation and showed that superior efficacyof SSRIs in reducing incidence of xerostomia and constipation. Barkin *et al*. has detected that TCAs can induce a decrease in gut motility and salving flow, which might have a tight association with the incidence of xerostomia and constipation (19). Additionally, insomnia may be a side effect of some antidepressants such as TCAs and SSRIs, or may be related to depression. Our finding illustrates that there was no significant difference between TCAs and SSRIs in causing the incidence of insomnia. However, numerous studies have been demonstrated that low-dose of antidepressants can be used to treat insomnia(20). Vasar *et al*. has detected that 6 days of fluoxetine administration spurssignificant decrease in rapid-eyes-movement (REM)sleep and increase in sleep latency and REM latency, without a dramatically increase in the number of awakenings during the night (21). In our meta-analysis, all agents of TCAs and SSRIs were administrated at normal dose level with the mean follow-up of 17.6 weeks. Therefore, our studies confirm that the incidence of antidepressant-induced insomnia was highly correlated with the dosages of antidepressants. 

However, the results of this meta-analysis must be interpreted with some cautions since the intrinsic limitations exist. First, the number of studies with the same index selected in our study is too small due to the inclusion criteria, larger number of trials and newer drugs are still warranted in the future study. Secondly, most trials excluded patients with dementia and psychotic depression, which might lead to the selective bias. Last but not least,there is no comparison of subgroup of gender, age and dosage of agent in this meta-analysis, which might affect the final results.

## Conclusions

A total of 5 selected literatures achieved the high jaded score of 5. However, the synthesis of data is subject to the limitations imposed by the criteria set for inclusion in the review. The review included a broad range of study designs, diagnosis, treatment settings andclinical outcomes. Conducting meta-analyses across disparate study settings could be of concern. The results of the present meta-analysis indicate that SSRIs are superior to TCAs in reducing the incident rate of xerostomia and constipation among patients with PD-induced depression.Yet, there was no significant statistical difference in the response rate of treatment, HAMD score, the insomnia rate between two groups, whatever its reasons, should lead us to call for reconsideration of their use. Pending that, new and larger sample of trials will be selected in the future study.
